# Evaluation of natural sounds in urban greenery: potential impact for urban nature preservation

**DOI:** 10.1098/rsos.170037

**Published:** 2017-02-15

**Authors:** M. Hedblom, I. Knez, Å. Ode Sang, B. Gunnarsson

**Affiliations:** 1 Department of Forest Resource Management, Swedish University of Agricultural Sciences, 901 83 Umeå, Sweden; 2 Department of Ecology, Swedish University of Agricultural Sciences, PO Box 7044, 750 07 Uppsala, Sweden; 3 Department of Social Work and Psychology, University of Gävle, 801 76 Gävle, Sweden; 4 Department of Landscape Architecture, Planning and Management, Swedish University of Agricultural Sciences, PO Box 66, 230 53 Alnarp, Sweden; 5 Department of Biological and Environmental Sciences, University of Gothenburg, PO Box 461, 405 30 Gothenburg, Sweden

**Keywords:** urban greenery, soundscape, bird song, urban woodlands, urban planning, biodiversity

## Abstract

Most humans now live in cities and their main experience of nature is through urban greenery. An increasing number of studies show the importance of urban green spaces for well-being, although most of them are based on visual perception. A questionnaire examining people's evaluations of natural sounds was answered by 1326 individuals living near one of six urban green areas of varying naturalness in the city of Gothenburg, Sweden. Women and the elderly reported greater calmness when hearing bird song and rustling leaves (and placed a higher importance on the richness of bird species) than did men, younger and middle-aged individuals. Independent of age and gender, urban woodlands (high naturalness) had higher evaluations than parks (low naturalness). Our results suggest that to increase positive experiences of urban green areas, demographic variables of gender and age should be taken into account, and settings that mimic nature should be prioritized in planning.

## 1. Introduction

More than 54% of the global population live in cities and the urban population is predicted to increase from the current 3.5 billion to 6.2 billion in the next 30 years [1,2]. Thus, most people on Earth now have their daily contact with nature and wildlife through urban green spaces. Contact with or perception of urban green spaces seems to have beneficial health effects, including shorter rehabilitation after surgery [3], increased well-being [4,5], reduced stress [6–10] and general increased happiness [11]. However, most studies on urban greenery and human well-being are linked to visual perceptions, despite the fact that humans perceive their environment using all their senses, including sight, smell and hearing. Given this, we investigated how natural sounds or soundscape ecology [12] influence people's experience of urban greenery. In urban areas, constant traffic noise can cause severe sleep disturbance, raised stress levels, increased blood pressure and heart disease [13]. In larger European cities, half the population is likely to be exposed to noise levels that can affect their well-being and health (The European Directive COM 2002/49/EC). Urban green areas, however, seem both to reduce noise exposure and provide citizens with an area of potential peace [14]. However, no area is completely silent (even remote natural areas) and in cities, urban natural sounds and noise are constantly intermixed. Pijanowski *et al*. [12] state that a new approach to identify high quality sounds with positive benefits and associations for humans is needed to enable conservation strategies for sound. They [12] also emphasize that soundscapes provide a research opportunity for linking human and environmental interactions, through, for example, spatio-temporal acoustic patterns, soundscape conservation and ecosystem monitoring.

Natural sounds, like natural sights, have been shown to reduce stress [15] but in addition visual settings and sound are strongly linked [16]. Viollon *et al*. [16] showed that the more urban a visual setting, the more negative the sound ratings. However, this influence depended on the type of sound where, for example, bird song was lower rated in strongly urban visual settings. In a study with a virtual reality forest, it was shown that there were significant differences in physiological recovery between test situations with and without sounds of nature [17]. Sounds are important for experiencing settings and in both [17] and [18] test participants were not comforted when exposed to nature photos (or VR-environments) without sound. Thus there seems to be an interaction between the visual and acoustic environments, and neither dominates. Green spaces are often not further defined, being simply described using terms such as ‘nature’, ‘green’ or ‘green infrastructure’, thus not differentiating between parks or natural habitats [19]. Similarly, sounds that reduce stress or increase well-being are often vaguely described using terms such as ‘natural sounds’ or ‘twittering birds’ or ‘sounds of light breeze’ (e.g. [15–17,20,21]). However, Ratcliffe *et al*. [22] counted frequency of mention per sound type across all participant interviews, where sounds were mentioned in relation to stress recovery or attention restoration. Bird song emerged as the type of sound most frequently mentioned in this context followed by sounds of water, non-avian animals, elements (e.g. soft wind and rain), and other sounds such as interactions with nature and silence. Hedblom *et al*. [23] further revealed that not only did bird song in general increase positive ratings of photos of urban settings, but when more species sang the ratings were higher. Their study [23] indicated that ‘heard biodiversity’ *per se* may have effects on our perceptions of urban areas.

Where there are positive experiences to natural sounds in urban environments, it would be interesting to examine associations with demographic variables such as age and gender. Several studies have reported on gender as well as age differences with regards to amount of activities, aesthetic ratings of natural environments and associated health and well-being [24–28]. Ode Sang *et al*. [24] reported that women were more active in green spaces compared with men, gave higher aesthetic ratings and also had a higher well-being associated with a higher activity for people's local green space. The same study also showed that older residents gave a greater aesthetic value and associated a higher well-being to their local green space compared with younger people. The studies by Richardson and Mitchell [25], de Vries *et al.* [26] and Astell-Burt *et al*. [27] showed differences in the associated health benefits with regard to gender as well as age, suggesting that the health benefit of closeness to urban green space varies between women and men and also during the lifespan of people. Explanations for these differences have been suggested to be associated with variation in increased time spent in nearby nature of groups such as elderly and housewives [26]. However, there is a need for further studies into the characteristics of these differences in relation to people's experience in urban green space. One aspect that presently is lacking, as far as we know, is how such demographic variables are linked to perceptual valuations of different natural sounds in urban green areas. The aim of the present study is to examine how people evaluate their local urban green spaces associated with natural sounds. Specifically, we aimed to investigate how evaluations of natural sound differs between areas perceived as having ‘high naturalness’ and ‘low naturalness’ and whether these evaluations vary with gender and age. More precisely, we investigate effects of high-versus-low naturalness, gender and age on ratings of greenery-related sounds (or sounds heard in urban green areas) such as bird song, many species singing, natural sounds, rustling trees, noise from the city, human voices as well the importance of visual features such as ‘trees’ and ‘shrubs’ for perceptions of birds. We discuss our results in relation to the ongoing densification trend within cities, causing urban green areas to become fragmented or to be removed, and we highlight the potential for using and designing urban green areas to conserve natural sounds for human benefit.

## 2. Method

### 2.1. Overview of the design

**Figure 1.**
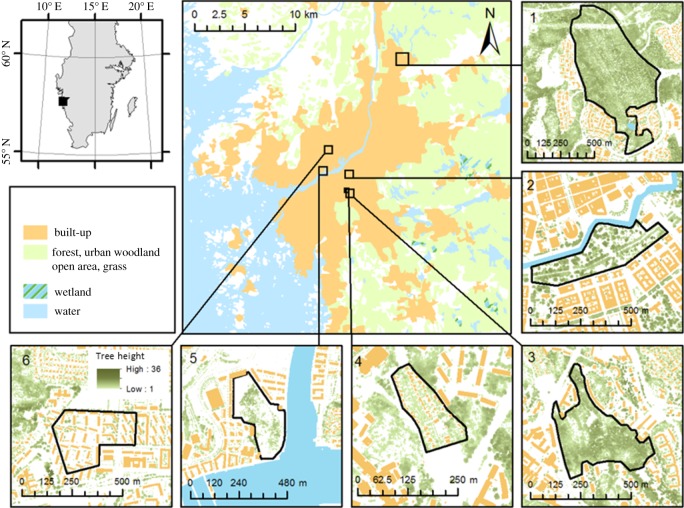
Location of the six different areas across the city of Gothenburg. (1) Suburban woodland (Titteridamm); (2) Old park (Kungsparken); (3) Urban woodland (Guldheden); (4) Allotment (Änggårdskolonin); (5) Park and woodland (Sörhallsparken); (6) Multifamily housing and lawn area (Wieselgrensplatsen). Adapted from Klingberg *et al*. [29].

**Figure 2.**
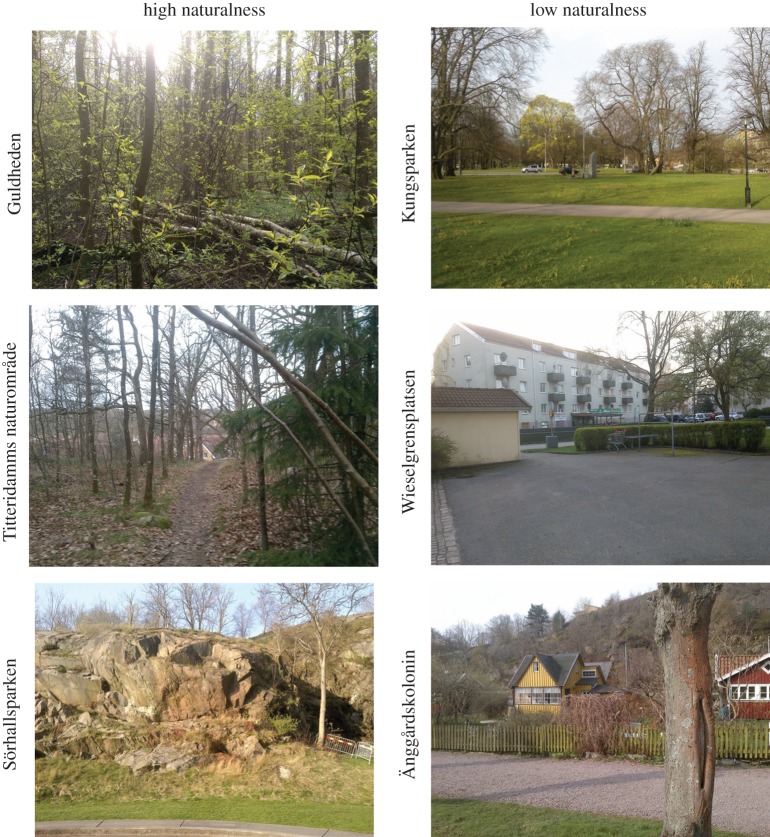
Photos of the six different areas that were considered to exhibit either high naturalness or low naturalness. Photos by Erik Heyman.

We selected six urban sites along an urban transect (from centre to urban fringe) in the city Gothenburg (approximate population is 530 000) in Sweden. The sites ranged from a residential area with lawn dominating to suburban woodland with predominantly natural vegetation (see study sites in figures 1 and 2). People living near or adjacent to the urban green areas were sent a survey relating to their experiences of that particular site.

### 2.2. Participants

A total of 2866 participants were sent a survey. They were randomly selected from a register of the population within postcode areas adjacent to the urban green spaces (see figure 1). The survey comprised a number of sections including questions about demographic variables, urban-greenery-related behaviour, experiences, perceptions and attitudes. After making contact three times, 1347 replies were obtained, involving 56.8% women and 43.2% men, distributed across six age groups of ≤25 (9.2%), 26–35 (24%), 36–45 (12.5%), 46–55 (14.4%), 56–65 (21.4%) and 66+ (18.3%). All respondents indicated that they were familiar with that specific area by answering, for example, specific questions about the number of times they visited the area. The survey was conducted in accordance with APA's (American Psychological Association) ethics code. That is, the participants were fully informed about: (1) the aim of our research, its procedures, benefits to society and especially to people living in a city nearby urban greenery, as well as the length of participation; (2) their right to withdraw from the study at any time without any consequences; (3) reasonable factors that may influence their willingness to participate, for example, how long it will take to complete the questionnaire and information about the types of questions included in the questionnaire; (4) confidentiality; (5); that they will not be financially compensated for participation; (6) whom to contact about any questions related to the study; and (7) that findings based on this survey will be reported in multiple publications. In this study we emphasize how age and gender relate to evaluations of different types of greenery, natural sounds and noise, involving 1326 participants (see table 1 and electronic supplementary material, file for all data used in analyses).

**Table 1.** Number of male and female participants across Naturalness and Age.

	naturalness		
	low	high	total
male			
age			
younger	102	88	190
middle-aged	86	75	161
older	89	135	224
total	277	298	575
female			
age			
younger	149	104	253
middle-aged	92	103	195
older	123	180	303
total	364	387	751
total			
age			
younger	251	192	443
middle-aged	178	178	356
older	212	315	527
total	641	685	1326

### 2.3. Study sites

The study sites were: (1) Suburban woodland (local name Titteridamm, size 38.4 ha) surrounded by a residential area with row houses and small buildings adjacent to a mixed coniferous and deciduous forest (*Betula pendula*, *B. pubescens*) with pine (*Pinus sylvestris*) and spruce (*Picea abies*) as the dominant species. (2) Urban woodland (local name Guldheden, size 12 ha) surrounded by three-storey buildings, taller tower blocks, a University hospital and local traffic routes. The site is dominated by deciduous trees such as oak (*Quercus robur*), aspen (*Populus tremula*) and birch. (3) Park and woodland (local name Sörhallsparken, size 6.4 ha) which is a newly established park with open lawns and a few small ornamental trees, including a rocky hill and an urban woodland in the middle. The park is surrounded by new multi-storey buildings on all sides except for one bordering the Göta river. (4) Allotment area (local name Änggårdskolonin, size 1.9 ha). The allotment was founded in 1913 and is situated between a university campus area and residential area of three-storey buildings. The allotments contain predominantly domesticated trees such as apple (*Malus × domestica*) together with multiple ornamental plant species. (5) Old park (local name Kungsparken; approximately 150 years old, size 9.8 ha) in the very centre of the city surrounded by an old canal and houses built in the late nineteenth century. Several busy traffic routes and paved walkways cross the park. Many trees are veterans, including specimens of oak, lime (*Tilia cordata*), beech (*Fagus sylvatica*) and several introduced species such as horse chestnut (*Aeculus hippocastanum*). The ground cover is mainly lawn. (6) Multifamily housing and lawn area (local name Wieselgrensplatsen, size 8.5 ha). The area is a mixture of three-storey buildings from the 1940s and includes small roads. Well-managed lawns dominate the courtyards and there are very few, mainly solitary, trees such as willow (*Salix fragilis*) and maple (*Acer* sp.)*.* See Gunnarsson *et al*. [30] for further details of the study sites.

### 2.4. Survey questions and responses

#### 2.4.1. Sound evaluations related to urban greenery

Participants were asked to evaluate (rate its desirability) different types of sounds using a seven-point scale, ranging from 1 (completely disagree) to 7 (completely agree). The measure comprised five statements, with a Cronbach's α of 0.81; indicating high reliability: ‘Sounds of nature give me a stronger perception of the site’; ‘Rustling trees make me feel calm’; ‘Bird song in the area makes me feel calm’; ‘Human voices make me feel calm’; and ‘Noise from the city and traffic interferes with my perception of the area’ (see also [24] and [30]).

#### 2.4.2. Bird song evaluations

Participants were asked to evaluate (rate its desirability) the bird song in urban greenery on a seven-point scale, ranging from 1 (completely disagree) to 7 (completely agree), with a Cronbach's α of 0.93, involving two statements: ‘It is important for me to hear birds singing in the area’; and ‘It is important for me to hear the songs of many bird species in the area’.

#### 2.4.3. Importance of nature-related sounds, trees and plants for the experience of bird species

Participants were asked to respond to three statements on a seven-point scale, ranging from 1 (completely disagree) to 7 (completely agree), with a Cronbach's α of 0.95: ‘Sounds from nature are important for my experience of bird species in the area’; ‘Trees are important for my experience of bird species in the area’; and ‘Plants are important for my experience of bird species in the area’. Accordingly, in this task the participants were asked to value the importance of these three variables for the experience of bird song.

### 2.5. Design and analyses

A non-equivalent comparison-group quasi-experimental design was used [31]. This means that the participants were not randomly assigned to the different groups of naturalness and age and that these independent variables may have been confounded with some uncontrolled extraneous variables. Thus, compared with a ‘true experiment’ [32], the inferences that can be drawn about the causal relationships between independent and dependent variables could be considered to be weaker (see e.g. [33,34] for similar treatment of cross-sectional data).

#### 2.5.1. Independent variables

Three independent variables included were: two levels of Naturalness (high and low) × two genders (women and men); × three Age categories (younger, middle-aged, and older). This means that the green spaces were redefined from six to two ‘perceived naturalness’ categories of high versus low, based on the mean value of the different perceptual categories evaluated by the respondents. Areas classified here as being of high naturalness do not *per se* have, for example, a higher diversity of species or higher densities of trees than an area of low naturalness but they are *perceived* as having higher naturalness (see figure 2 for photos of the areas). Areas considered to exhibit ‘high naturalness’ were the two ‘urban woodland’ areas (Titteridamm and Guldheden) and one ‘park and urban woodland’ (Sörhallsparken) while the areas considered to exhibit ‘low naturalness’ were ‘old park’ (Kungsparken), ‘allotment’ (Änggårdskolonin) and the ‘multifamily housing and lawn area’ (Wieselgrensplatsen). In addition, the six age categories were reclassified into three groups of younger (≤25–35), middle-aged (36–55) and older (56–66+) participants. We dichotomized naturalness and trichotomized age because there were too few respondents in Gender by Naturalness by Age cell-combinations (see table 1 for the number of respondents in gender × naturalness × age cells).

#### 2.5.2. Dependent variables

Participants' evaluations of ‘greenery-related sounds’, ‘bird song’, ‘importance of nature-related sounds’ and ‘trees and plants for the experience of bird species’ were treated as dependent variables.

#### 2.5.3. Statistical analyses

Multivariate analysis of variance (MANOVA) was used because all three measures (dependent variables) contained more than one scale/dimension, measuring the latent variables of ‘greenery-related sound’ *desirability*, ‘bird song’ *desirability*, and *valuation* of the importance of ‘trees and plants for the experience of bird species’. Furthermore, we used Fisher's least significant difference (LSD) as *post hoc* test. The independent variables of Naturalness, Gender and Age were treated as between-subject factors. The software IBM SPSS Statistics 22 was used for statistical computations.

## 3. Results

The results are reported in three sections, related to the three dependent variables of sounds: ‘natural sounds’, ‘importance of bird song’ and ‘importance of sounds from trees and plants for the experience of bird species’. Only significant results are presented.

### 3.1. Natural sounds

**Figure 3.**
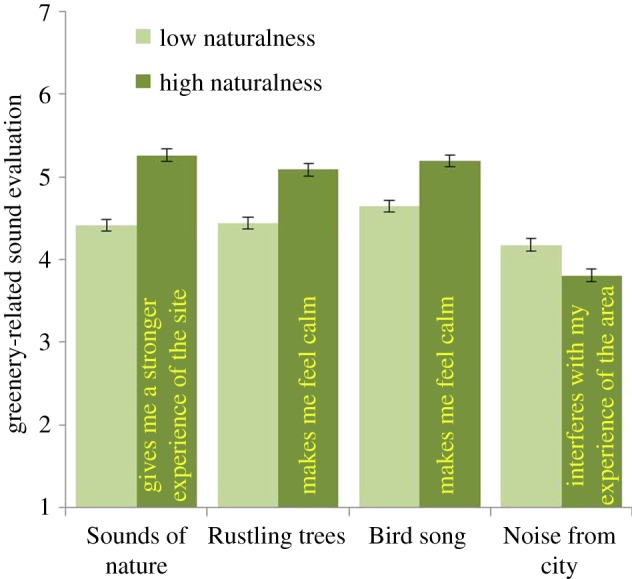
Effect of how respondents evaluate different sounds in areas with high naturalness (urban woodlands) or low naturalness (e.g. parks). The sounds were ‘Sounds of nature give me a stronger perception of the site’, ‘Rustling trees make me feel calm’, ‘Bird song in the area makes me feel calm’ and ‘Noise’. The columns illustrate mean values ± s.e. of dependent variables.

MANOVA showed three main effects of Naturalness, Gender and Age. The impact of Naturalness, Wilks *λ* = 0.91, *F*_5, 1211_ = 23.39, *p* < 0.01, *η*^2^ = 0.09, was associated with four statements: *Sounds of nature* give me a stronger experience of the site (*p* = 0.01); *Rustling trees* make me feel calm (*p* = 0.01); *Bird song* in the area makes me feel calm (*p* = 0.01); and *Noise* from the city and traffic interferes with my experience of the area (*p* = 0.01). As can be seen in figure 3, ‘rustling trees’ and ‘bird song’ elicited higher calmness in participants in areas with high naturalness than areas with low naturalness. Residents experienced more ‘noise’ in areas with low naturalness than in areas with high naturalness. Thus, the visual experience (high or low naturalness) was linked to the sound experience.

**Figure 4.**
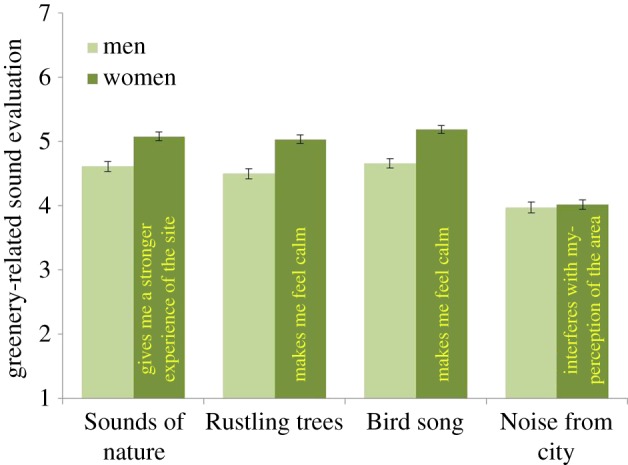
Effect of how different genders evaluate the sounds of nature, rustling trees, bird song and noise from the city. The columns illustrate mean values ± s.e. of dependent variables.

The impact of Gender, Wilks *λ* = 0.97, *F*_5, 1211_ = 6.77, *p* < 0.01, *η*^2^ = 0.03, was associated with four statements: *Sounds of nature* give me a stronger experience of the site (*p* = 0.01); *Rustling trees* make me feel calm (*p* = 0.01); *Bird song* in the area makes me feel calm (*p* = 0.01); *Human voices* make me feel calm (*p* = 0.01). The results suggest that women evaluated the urban-greenery-related sounds as being more positive than did men, but also voices were experienced as being more calming by women (see figure 4).

**Figure 5.**
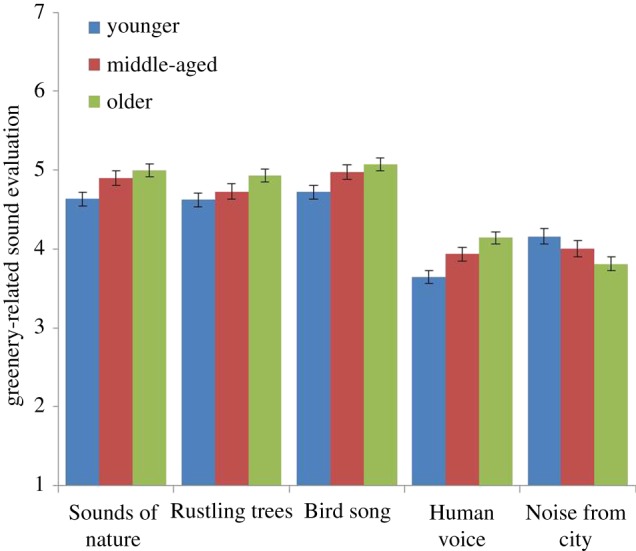
Effect of how people with different age evaluate the sounds of nature, rustling trees, bird song, human voices and noise from the city. The columns illustrate mean values ± s.e. of dependent variables.

The impact of Age, Wilks *λ* = 0.97, *F*_5, 1211_ = 3.62, *p* < 0.01, *η*^2^ = 0.02, was associated with all five statements: (1) *Sounds of nature* give me a stronger experience of the site (*p* = 0.01); (2) *Rustling trees* make me feel calm (*p* = 0.03); (3) *Bird song* in the area makes me feel calm (*p* = 0.01); (4) *Human voices* make me feel calm (*p* = 0.01); and (5) *Noise* from the city and traffic interferes with my perception of the area (*p* = 0.03). As can be seen in figure 5, positive experience of all types of urban-greenery-related sounds increased with age except for urban noise, which, in contrast, decreased with age. However, *post hoc* tests (LSD) showed that (statements 1–5): (1) no significant difference between middle-aged and older was indicated (*p* = 0.44); (2) only significant difference was shown between younger and older (*p* = 0.01); (3) no significant difference between middle-aged and older was indicated (*p* = 0.42); (4) no significant difference between middle-aged and older was indicated (*p* = 0.08); (5) only significant difference was shown between younger and older (*p* = 0.01).

### 3.2. Importance of bird song

**Figure 6.**
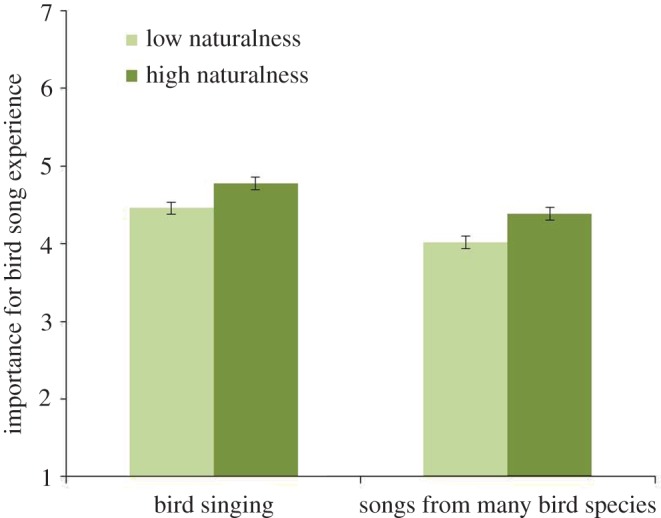
Effect of how respondents evaluate different sounds in areas with high naturalness (urban woodlands) or low naturalness (e.g. parks). The sounds were ‘birds singing’ and ‘songs from many bird species’. The columns illustrate mean values ± s.e. of dependent variables.

MANOVA showed three main effects of Naturalness, Gender and Age. The impact of Naturalness, Wilks *λ* = 0.99, *F*_2, 1240_ = 7.61, *p* < 0.01, *η*^2^ = 0.01, was associated with both statements: It is important for me to listen to the *birds singing* in the area (*p* = 0.01); and it is important for me to listen to *songs of many bird species* in the area (*p* = 0.01). As can be seen in figure 6, both ‘birds singing’ and ‘songs of many bird species’ were considered more important in areas with high naturalness than areas with low naturalness. Thus, the visual experience (high or low naturalness) was linked to the sound experiences in a similar way that was reported in §3.1.

**Figure 7.**
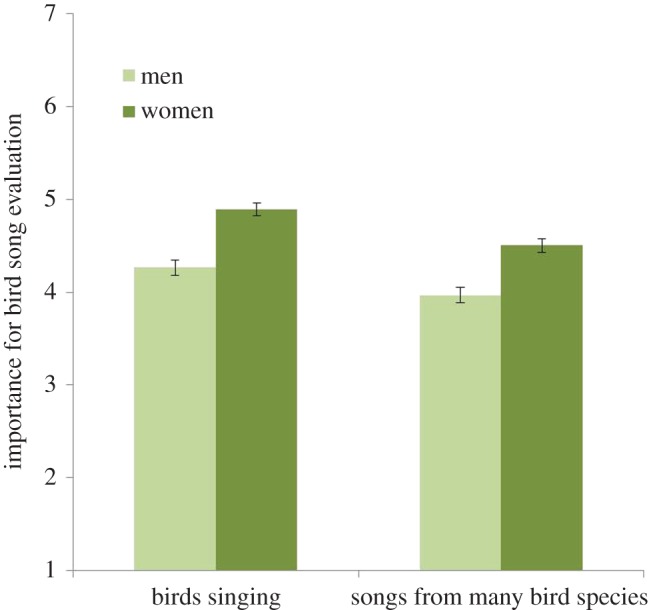
Effect of how different genders evaluate the sounds of ‘birds singing’ and ‘songs from many bird species’. The columns illustrate mean values ± s.e. of dependent variables.

The impact of Gender, Wilks *λ* = 0.98, *F*_2, 1240_ = 16.12, *p* < 0.01, *η*^2^ = 0.03, was associated with both statements: It is important for me to listen to the *birds singing* in the area (*p* = 0.01); and it is important for me to listen to *songs of many bird species* in the area (*p* = 0.01). For women compared with men, birds singing and the songs of many bird species in urban greenery were shown to be more important (see figure 7).

**Figure 8.**
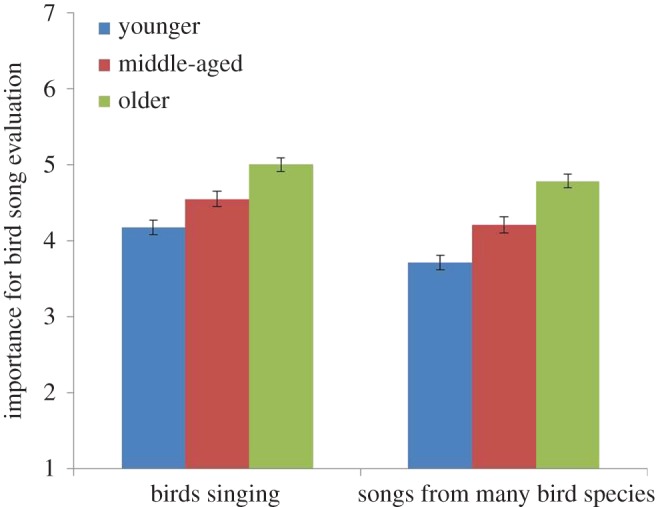
Effect of how people with different age evaluate the sounds of ‘birds singing’ and ‘songs from many bird species’. The columns illustrate mean values ± s.e. of dependent variables.

The impact of Age, Wilks *λ* = 0.95, *F*_2, 1240_ = 16.27, *p* < 0.01, *η*^2^ = 0.03, was associated with both statements: It is important for me to listen to the *birds singing* in the area (*p* = 0.01); and it is important for me to listen to *songs of many bird species* in the area (*p* = 0.01). As can be seen in figure 8, the importance of bird song *per se* in urban greenery was shown to increase with age. *Post hoc* tests showed that significant differences between age groups were obtained in all combinations (from *p* = 0.000 to *p* = 0.01).

### 3.3. Importance of sounds from trees and plants

MANOVA showed three main effects of Naturalness, Gender and Age. The impact of Naturalness, Wilks *λ* = 0.98, *F*_3, 1214_ = 7.17, *p* < 0.01, *η*^2^ = 0.02, was associated only with the statement *Sounds from nature* are important to my experience of bird species in the area (*p* = 0.01); showing that sounds from nature were more important for the experience of bird species in areas of high (*M* = 4.75, SD = 1.94) versus low (*M* = 4.3, SD = 1.99) naturalness.

**Figure 9.**
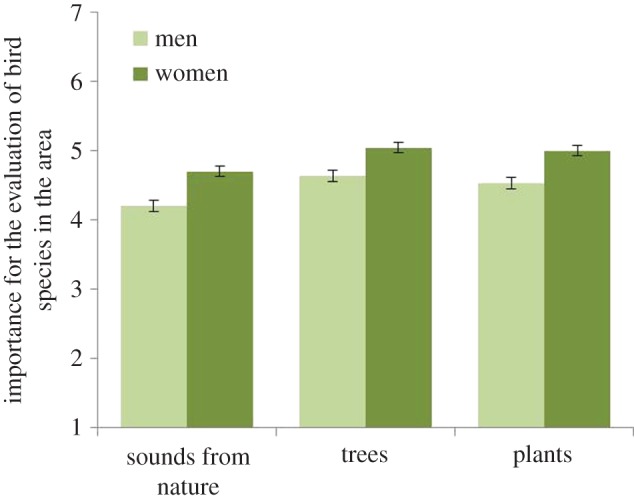
Effect of how different genders evaluate the importance of ‘sounds from nature for the experience of bird species in the area’, ‘trees for the experience of bird species in the area’ and ‘plants for the experience of bird species in the area. The columns illustrate mean values ± s.e. of dependent variables.

The impact of Gender, Wilks *λ* = 0.98, *F*_3, 1214_ = 7.64, *p* < 0.01, *η*^2^ = 0.02, was associated with all three statements: *Sounds from nature* are important for my experience of bird species in the area (*p* = 0.01); *Trees* are important for my experience of bird species in the area (*p* = 0.01); and *Plants* are important for my experience of bird species in the area (*p* = 0.01). For women compared with men, sounds from nature, trees and plants in urban greenery were all more important for the experience of bird species in urban greenery (see figure 9).

**Figure 10.**
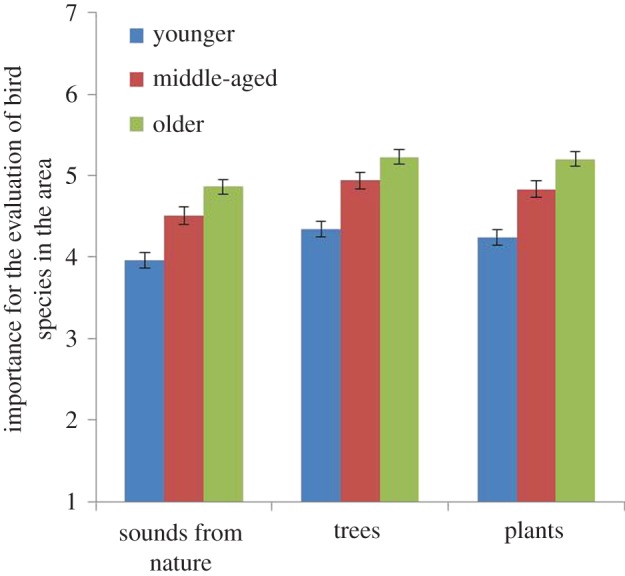
Effect of how respondents with different ages evaluate the importance of ‘sounds from nature for the experience of bird species in the area’, ‘trees for the experience of bird species in the area’ and the importance of ‘plants for the experience of bird species in the area. The columns illustrate mean values ± s.e. of dependent variables.

The impact of Age, Wilks *λ* = 0.95, *F*_3, 1214_ = 9.67, *p* < 0.01, *η*^2^ = 0.02, was also associated with all three statements: *Sounds from nature* are important for my experience of bird species in the area (*p* = 0.01); *Trees* are important for my experience of bird species in the area (*p* = 0.01); and *Plants* are important for my experience of bird species in the area (*p* = 0.01). As can be seen in figure 10, the importance of sounds from nature, trees and plants for the experience of bird species in urban greenery all increased with age. *Post hoc* tests (LSD) showed that significant differences between age groups were obtained in all combinations (from *p* = 0.000 to *p* = 0.03).

## 4. Discussion

Our results showed that areas with perceived *high naturalness* (e.g. urban woodlands) were linked to more positive nature-sound-related evaluations and higher importance for experiencing bird songs than in areas with perceived *low naturalness* (parks, allotments and lawns). It should be noted, however, that this difference partly could be influenced by the environment-related attitudes of people living nearby our study areas [30]. Independently of the type of naturalness in the urban green areas, women evaluated natural sounds and bird song higher than men did. The evaluations with respect to natural sounds and bird song *per se* (independent of naturalness) increased with age. Our results also indicate that visual and audible experiences of urban greenery are connected, in that areas with different naturalness (visual) had different evaluations of sounds, something that is missing in most of the studies of visual perception of urban green spaces and visual urban green planning.

No previous study has combined the evaluations of different qualities of urban greenery with natural sounds as we have in the current work. Here, areas with perceived high naturalness were related to the feeling of calmness engendered by natural sounds (sounds of nature, rustling trees, bird song; figure 3). Further, areas with high naturalness were reported as being more important to ‘bird song’ and ‘many birds singing’ experience than areas with lower naturalness (figure 6) did. These differences between urban green spaces are noteworthy since they reveal that expectations associated with perceiving natural sounds are greater in natural areas without intensive management than parks and designed areas.

If there is a higher expectation of finding natural sounds calming and if it is more important to experience bird song in areas that are more natural than parks, for example, this needs to be taken into consideration in both management and conservation of urban green areas when planning for recreation. At present many studies do not differentiate between the qualities of different urban green spaces and their impact on health (e.g. there are vague descriptions of what urban greenery actually consists of trees, shrubs, species etc. e.g. [35]) although such qualities may influence perceptions of green spaces and by that also health; but see other studies, such as Schipperijn *et al*. [36] (eight different urban green areas), Tyrväinen *et al*. [10] (urban, urban woodland and park) and Sonntag-Öström *et al*. [37] (forests). From a conservation perspective, natural areas in cities such as urban woodlands are less formal with respect to planning than, for example, designed parks, and thus are at higher risk of loss when new housing is needed. If so, the areas in this study that were linked to higher calmness evaluations and gained higher overall evaluations are potentially at higher risk of being destroyed or fragmented. Further, the existing woodlands in cities are often managed to reduce risks of falling branches and deadwood, turning them into areas more akin to parks than true natural settings [38] and thus reducing their naturalness.

In our case, the park-like areas (with low naturalness) have more roads, fewer shrubs and fewer trees than the urban woodlands (high naturalness), potentially making them more exposed to external sound sources [14] which may also affect people's evaluations of natural sounds. Although cities such as Stockholm (Sweden) may have high urban woodland cover (>20%), each individual woodland may be very small and may not provide a better situation for promoting natural sounds than cities that have lower cover such as Malmö (Sweden) with <2%. In Sweden and Denmark less than 50% of the existing urban woodlands are smaller than 2 ha [39]. In this study the average size of an area with high naturalness was 19 ha compared with the average size of areas with low naturalness of 7 ha (although we did not separate greenery from roads, water, hills and houses; see figure 1).

Stott *et al*. [40] showed that noise reduction increased with extent of urban greenery and they recommended retaining larger green areas in cities. It is, however, also possible to create barriers to block noise and promote natural sounds in smaller as well as larger urban green areas, as suggested by Slabbekoorn and Ripmeester [41]. They suggest that installing a solid barrier as close as possible to the noise source, adding an overhang on the side of roads or increasing the height of an existing barrier is a cost-efficient way to benefit pedestrians at ground level [37]. Moreover, such barriers could be combined with greenery and provide additional positive effects as habitats for birds [42].

Evaluations of natural sounds differ by gender in a similar way to visual perceptions of urban greenery (see [24]). In the current study, women evaluated all natural sounds higher than did men and they had a lower tolerance for noise than men (figure 4). Women reported that it was more important for them to hear bird song in their local urban greenery than did men (figure 7). For women, sounds from nature, trees and plants were all more important for experiencing bird species in urban greenery than for men (figure 9). Further, women reported that it was more important for them to hear many species (figure 6). So, why do we find these differences? In relation to explaining the gender differences that occurred in relation to health benefits, de Vries *et al*. [25] suggest that it could be explained by the time spent at home, and hence women and elderly would appreciate and value the nearby urban green space more. By spending more time within the area, the appreciation of bird song and its variation might increase and also an experience of a linkage between features such as trees and flowers with the prevalence of birds. Other suggestions for recorded differences are related to the perception of safety in nature, for which women showed a greater concern [43]. This could provide one explanation for the finding in the current study in which women evaluated all natural sounds higher than did men and they had a lower tolerance for noise than men (figure 4).

Nevertheless, assuming that people *can* detect differences in biodiversity and species richness linked to sounds, urban planners need to take into consideration that habitats in cities not only need to be large and reduce noise but may need to have characteristics that enable many species to thrive in them. At present the resources, in general, required by birds in cities are still available and of sufficient quality to enable people to hear and see many bird species, but it is predicted that these resources will decrease in the future through habitat fragmentation [44,45]. In general women are more likely to support wildlife and management to increase biodiversity then men are [46,47], although they are, as yet, underrepresented in decisions concerning conservation and safeguarding biodiversity globally [48]. Thus, the gender perspective should be applied when planning for nature conservation in cities. It has been suggested that there are differences in brain activity between women and men when listening to music [49] which may be relevant also when natural sounds are perceived. Why women evaluate natural sounds more strongly remains partly unanswered.

Urban natural sounds were more important for older people than middle-aged and younger people. Older people reported having a stronger experience of the site related to the sounds of nature, and felt calmer as a result of the sound of rustling trees and bird songs than middle-aged and younger people (figure 5). Our results may indicate that older people spend more time during their life in their local urban greenery than middle-aged and younger individuals and thus evaluate these areas higher. And it could be that larger shares of the older population having grown up in more natural settings, and thus having higher appreciation of nature [50]. The psychological theory of place-identity reveals that a physical place can act as a reminder of important and collective experiences and identifications and thereby increase perceptions of local areas [51–53]. This is in line with Dzhambov and Dimitrova's [54] findings, which revealed that time and frequency of visits to a park decreased health anxiety among the elderly. In planning, there are numerous studies showing that people prefer to use urban greenery closest to where they live [55] and that reduced distance to urban green spaces is strongly linked to improved health [56]. Short distances to greenery may, in the case of the elderly (and children), be of even greater importance since they probably use neighbourhood woodlands and parks more than the more mobile younger and middle-aged groups. A combination of distance to and quality of urban greenery is the main reason for people to use such spaces [36].

The elderly reported that it was more important to listen to bird songs in the areas studied and that it was more important to hear more species singing (figure 8) than did middle-aged or younger people. Further, the elderly found natural features such as trees and plants to be important for experiencing birds in the area (figure 10). In line with this, Shwartz *et al*. [57] and Belaire *et al*. [58] showed that older people had more positive perceptions of birds than younger people and that they were more willing to spend money on feeding birds [59]. Another study [60] found differences in positive perceptions of species *per se*: older Norwegians rated ‘small birds’, ‘seagulls’ and ‘magpies’ higher than ‘birds of prey’ and these ratings declined with respondents' age. Younger people in our study, however, were less tolerant of noise than older people, and felt less calmed by human voices than did older people (figure 5).

Reducing noise in cities not only increases people's opportunities to hear natural sounds but also increases the chance that birds, for example, will thrive. At present birds (like humans) avoid areas with high noise levels [41] or change their behaviour, so that they may sing much earlier in the mornings to avoid traffic and, consequently, few people are up to hear them singing [61]. There is rather rapid evolution of birds in urban areas, which sing louder and at a higher pitch than rural birds [62]. How this may affect humans is not known. Hedblom *et al*. [23] showed that the more complex and ‘melodious’ song of the willow warbler (*Phylloscopus trochilus*) was preferred over the chattering of house sparrows (*Passer domesticus*), and Ratcliffe [63] showed that that smooth or consonant sounds were considered more pleasant than rough sounds.

Future studies should link the tones of natural sounds to specific species or diversity of species and not simply as coarse types of sounds as we did in this study. People are individuals and have different associations to specific bird species’ sounds [64]. Further studies should also deepen the knowledge of how to manage urban greenery for increased visual and sound experiences in relation to gender and age, thus also include children, who were not studied here. Finally, it would be preferable to compare how people perceive natural sounds in relation to different noise interferences (such as traffic) in order to reveal if there is a threshold between acceptable noise interference and no interference. This could then be referred to the size of the urban green space, damping effect by trees and shrubs or the need for artificial noise barriers (e.g. green walls [42]).

The results reported herein are applicable to non-urban areas as well, maybe even more so in some cases since people do expect to experience solitude and silence more in remote places and pristine nature than in urban areas. Thus, the age and gender perspective, including access and availability, presumably should be taken into consideration in areas promoting tourism.

When most people on Earth now spend their lives in urban areas and reportedly visit the green spaces close to their homes more often than remote pristine nature, the quality of these experiences in terms of sight and sound (and smell) potentially may be important for their connection to and understanding of nature away from cities [65–67]. Thus, it is important to create urban green areas that allow people not only to perceive landscapes visually, but also through sound.

## 5. Conclusion

Most literature describing responses to urban greenery comes from visual studies, even though humans perceive their environment with all their senses. In the present study we have shown that people evaluate natural sounds differently depending on gender and age but also according to whether the sound is heard in an area with high naturalness. Women and the elderly had stronger responses and experienced greater calmness as a result of hearing natural sounds than did men and younger and middle-aged people. Women and the elderly also found it more important to hear the songs of many species of birds and for urban green spaces to contain tree and plants, than did men and younger and middle-aged people. In general, people reported higher evaluations of natural sounds in areas that they considered being highly natural, such as urban woodlands compared with less natural areas, such as parks, allotments and lawns.

These results, demonstrating greater reported benefits of natural areas than parks, have implications for the need to conserve the most natural areas in cities. At the same time as an increasing number of studies reveal greater value to humans of urban greenery, such areas are rapidly becoming fragmented and declining globally. Newly established areas are dominated by parks with a field layer comprising monoculture lawns rather than more natural habitats. Thus, conserving urban woodlands with low noise from surrounding city activities would be of great value, especially for the elderly and for women.

## Ethics

We followed the guidelines for good research ethics as recommended in booklet ‘Good research practice, Swedish research council 3:2011’ where it is emphasized that the participants should be informed that they are participating in a research project. Through this we also followed the ethical guidelines of the University of Gothenburg in charge of this project. According to the Swedish ethical guidelines (‘Good research practice, Swedish research council 3:2011), a research project should be reviewed by an ethics review board if any of the following conditions exist. Namely, if the project (A)

— entails physical encroachment on the research subject,
— will be conducted using a method aiming to affect the research subject physically or psychologically,
— carries an obvious risk of physical or psychological harm to the research subject, or
— entails studies on biological material that can be traced to specific individuals.

A research project should also be reviewed if it (B)

— entails the handling of sensitive personal data according to 13 § of the Personal Data Act (SFS 1998:204), including information on race, ethnic origin, political views or religious conviction or personal data according to 21 § of the Personal Data Act, including information on judgements in criminal cases.
  (1) In our case we informed the participants that their answers will be used in a research project and treated the entire data totally anonymous (see below) and thus did not need any ethical review board.
  (2) We sent out the questionnaire to participants by ordinary mail using a professional firm specializing in questionnaires. The questionnaires were randomly sent to postboxes within areas in the vicinity of the urban green area studied. They were randomly chosen from a large number of households.
  (3) The participants were given information on the front page of the questionnaire explicitly stating that the answers from the participant will be used in a research project and that their contributions are of utmost importance for this project. Thus participation was entirely voluntarily.
  (4) The participants could either answer on the papers send to them or create an account with their own login passwords on the internet. The participants never wrote their names or addresses so that they could be traced.

## Supplementary Material

Data for table 1 and figure 3-10

## Data accessibility

The datasets supporting this article have been uploaded as part of the electronic supplementary material.
